# Beyond Coding: Balancing Interpretive Depth and Rigour in Ethnographic Data Analysis

**DOI:** 10.1111/jan.70682

**Published:** 2026-07-31

**Authors:** Thi Ngoc Minh Nguyen, Gordana Dermody, Rosemary Saunders, Lisa Whitehead

**Affiliations:** ^1^ School of Nursing and Midwifery Edith Cowan University Joondalup Western Australia Australia; ^2^ School of Health University of the Sunshine Coast Sunshine Coast Queensland Australia

## Abstract

**Aim:**

To present a refined approach to analysing ethnographic data that preserves the distinctiveness of ethnography while ensuring it meets contemporary expectations of methodological rigour.

**Design:**

Methodological discussion paper.

**Data Sources:**

The approach draws on established ethnographic scholarship and is guided by Wolcott's framework for transforming qualitative data through three interrelated stages: description, analysis and interpretation. A practical application is illustrated through a focused ethnography exploring how older Vietnam‐born Australians understand and manage chronic disease.

**Implications for Nursing Practice:**

Focused ethnography has become increasingly popular in nursing research as it reflects the dual nature of the profession: striving to generate rigorous evidence while preserving the humanistic depth and contextual understanding that are central to nursing practice. This paper provides a methodological guide for nurse researchers to analyse ethnographic data in ways that preserve contextual richness while ensuring analytical rigour.

**Impact:**

The approach presented advances the debate on the methodological quality of research in nursing and health and supports the generation of high‐quality evidence.

**Conclusion:**

Ethnography, particularly in its focused form, must continue to meet high standards that balance art and science, narrative and analysis. Nurse researchers are encouraged to embrace a balanced approach to ensure that ethnography remains both scientifically credible and deeply human.

**Patient or Public Contribution:**

Patients or the public were not involved in this methodological discussion paper; the illustrative example draws on ethically approved doctoral research.

## 1 Introduction

In the early 2000s, focused ethnography was formally introduced as a distinctive methodological approach characterised by a well‐defined research focus and short‐term intensive observations (Knoblauch 2005). Focused ethnography has been embraced across disciplines, including medicine, nursing, health, social sciences and education (Trundle and Phillips 2023). Focused ethnographic research aims to provide in‐depth, contextually rich insights into the complexities of healthcare systems, patient experiences and healthcare practices within specific communities or settings. By capturing qualitative aspects such as cultural beliefs, patient–provider interactions and social determinants of health, focused ethnography contributes to the development of culturally sensitive interventions and healthcare practices (Black et al. 2021).

Notwithstanding its popularity, the ways in which researchers practise focused ethnography, particularly in relation to ethnographic data analysis, have raised considerable methodological concern (Trundle and Phillips 2023). The introduction of this newer form of ethnography, the focused one, has not always been accompanied by thorough methodological preparation, particularly in relation to data analysis. In contemporary practice, ethnographic data are often analysed using common qualitative methods such as thematic analysis, content analysis, discourse analysis, or constant comparison (Chesnay 2015; Roper and Shapira 2000). While each of these methods carries distinctive features, they share the foundational aim of aggregating large volumes of qualitative data into categories, subcategories and themes that respond to research questions. On one hand, these structured approaches provide a level of transparency and systematic rigour that strengthens the credibility of qualitative research. On the other hand, they risk reducing the interpretive and contextual richness that is central to ethnography. This tension has prompted us to reflect critically on current analytical practices. We wonder: does the rapid, theme‐focused analysis of data lead to an oversight of crucial elements within ethnographic research? Such limitations are particularly pronounced in applied fields like nursing, health and social sciences, where researchers operate under time constraints and are expected to balance between analytic rigour and interpretive depth, and between emic and etic voices. Yet guidance on analysing ethnographic data in this way remains limited.

This paper advances methodological discussion in focused ethnography by clarifying the analytic commitments that underpin ethnographic data analysis and examining the limitations of relying on single, procedural techniques. We synthesise contemporary concerns regarding systematisation, digital contexts and the potential erosion of interpretive depth, and argue that analytic rigour in ethnography must be understood as both scientific and humanistic. In response, we reposition Wolcott's (1994) framework of description, analysis and interpretation as a structured yet interpretively grounded approach capable of preserving contextual integrity while ensuring methodological transparency. By integrating this framework with Richardson's (2000) evaluative criteria, we offer a coherent articulation of how focused ethnographic analysis can achieve both interpretive depth and scientific credibility.

## 2 Re‐Thinking Ethnographic Data Analysis

### 2.1 Current Practices and Limitations

The evolution of ethnographic data analysis reflects broader epistemological shifts in social research from early descriptive traditions to more systematic and reflexive approaches. This ongoing movement always represents a negotiation between interpretive understanding and scientific rigour.

Early ethnographic work was grounded in long‐term field engagement and narrative interpretation, with meaning‐making and the balance between emic and etic perspectives central to inquiry (Boas 1920; Malinowski 1922). Since the 1970s, qualitative inquiry has moved toward more systematic and structured analytic procedures (Miles and Huberman 1994; Glaser and Strauss 1973). In contemporary focused ethnography, analysis is commonly described as a process of identifying, categorising and organising data into patterns and themes (Andreassen et al. 2020; Higginbottom et al. 2013; Vindrola‐Padros 2021). In focused ethnography, methodological guides and applied studies frequently describe analysis as a process of identifying, categorising and organising data to abstract patterns and themes. Saldaña (2016) work on coding has influenced many contemporary ethnographers, showing how systematic techniques can be integrated with interpretive depth. While these approaches enhance transparency and analytic order, they also introduce a risk: rich, contextual data may be fragmented into decontextualised categories, reducing ethnography's interpretive depth.

These tensions are increasingly visible across contemporary forms of ethnographic research. The emergence of digital ethnography has expanded the contexts in which social life can be studied while raising questions about the nature of ethnographic engagement (Burrell 2009). Although online environments generate large volumes of accessible data, data abundance does not necessarily translate into interpretive depth. Rather than evaluating these developments, this paper emphasises a broader methodological principle: regardless of context, ethnographic analysis must remain consistent with its epistemological commitments, preserving the relational, contextual and interpretive grounding that defines ethnographic inquiry (Angelone 2019; Forberg and Schilt 2023).

The shift toward increasingly technical and standardised approaches to ethnographic data analysis has generated sustained concern. Scholars have cautioned that such proceduralization may dilute ethnography's distinctive identity and make it more difficult to sustain its interpretive standards (Marcus 2006; Mendenhall et al. 2016). While structured approaches enhance transparency, they may fail to convey the authenticity and contextual richness of field experience, where narrative remains central to ethnographic evidence (Forberg and Schilt 2023). Without this alignment, ethnography risks becoming a form of distant observation rather than lived understanding. This highlights the importance of maintaining coherence between analytic practices and the epistemological commitments of ethnographic inquiry, particularly in preserving interpretive integrity, reflexivity and contextual grounding.

### 2.2 The Commitments of Ethnographic Data Analysis

Understanding the commitments of ethnographic data analysis requires asking a fundamental question: what does ethnographic analysis commit to, and why might it require more than a single analytic technique? Addressing this question begins with recognising the distinctive nature of ethnography. Ethnography refers both to a process of learning about people in their natural settings and to an interpretive product: the written account that represents their lived experiences within social and cultural contexts (Atkinson 2014; Boyle 1994). Regardless of its form: traditional, focused, or rapid, ethnography is defined by its attention to relational, contextual and multilayered data. Crucially, these features are not preserved automatically through fieldwork; they are realised through analytic practice. It is in the process of data analysis that ethnography's distinctive nature is either maintained or diminished (Marcus 2006).

If ethnographic analysis is committed to preserving contextual depth while meeting expectations of rigour, then its evaluation must reflect these commitments. Ethnography cannot be judged solely through procedural criteria borrowed from other qualitative traditions. As Richardson (2000) reminds us, it must be held to ‘high and difficult standards’, evaluated on both scientific and humanistic grounds. Rigour in ethnography therefore extends beyond transparency or coding procedures to include interpretive integrity, reflexivity, narrative coherence and contextual grounding. Because ‘Without science, we lose our credibility. Without humanity, we lose our ability to understand others’ (Agar 1996, 13).

Ethnographic data analysis thus requires navigating these dual commitments, producing findings that are systematically developed while remaining faithful to lived experience (Richardson 2000; Wolcott 1994). This balance cannot be achieved through a single analytic technique. When ethnographic material is reduced to coding and themes alone, there is a risk of fragmenting relational meaning and weakening the interpretive depth that defines the methodology. Good ethnography remains grounded in narrative: not as anecdote, but as purposeful, theoretically informed and analytically structured account (O'Reilly 2015; Wolcott 1994).

The analytic challenge, therefore, is not whether to pursue rigour, but how to enhance it without eroding interpretive depth (Johnson et al. 2020). This requires an approach that maintains coherence between analytic practices and the epistemological commitments of ethnography. In response, we draw on Wolcott's (1994) framework of description, analysis and interpretation as a structured yet flexible approach for ethnographic data analysis. This framework stages the movement from description to analysis and interpretation, enabling the integration of narrative and systematic meaning‐making while preserving contextual integrity. In the next section, we present Wolcott's framework and demonstrate how, when viewed through Richardson's (2000) evaluative criteria, it supports rigorous and interpretively grounded ethnographic analysis.

## 3 Wolcott's Framework: A Timely Analytic Guide for Focused Ethnography

In this paper, we re‐position Wolcott's framework as a structured analytic approach that enables researchers to preserve the interpretive depth distinctive to ethnography while ensuring methodological rigour in the analysis of ethnographic data. Wolcott (1994) proposed a framework for transforming qualitative data through three interrelated stages: description, analysis and interpretation. Rather than presenting these as isolated techniques, Wolcott conceptualised them as progressive transformations of ethnographic material. Wolcott's framework is not a recent methodological innovation; however, its enduring relevance lies precisely in its clarity regarding the staged movement of ethnographic inquiry. Rather than offering a technical coding procedure, Wolcott conceptualised analysis as a progressive transformation of data that preserves narrative integrity while enabling systematic meaning‐making. This distinction remains particularly timely in contemporary focused ethnography, where analytic processes are often compressed and structured under increasing expectations of procedural transparency.

### 3.1 Description

Wolcott's (1994) framework for transforming qualitative data begins with *description* as he believes that being *a good storyteller* in ethnography is as important as engaging in systematic meaning‐making. It is the process of ‘getting the story out’, of constructing a rich and coherent account that captures what was observed, experienced and recorded in the field before moving to more abstract interpretation. Description, for Wolcott, is not a simple act of reporting or summarising data. Description, rather, it is the first layer of analysis. Through this process the researcher presents the world of participants as faithfully and vividly as possible. In ethnographic work, description plays a crucial role in preserving the integrity and texture of field experiences, enabling readers to enter the participants' social world and grasp the meanings embedded in it.

The descriptive stage is particularly crucial in focused ethnography, where field engagement is typically short‐term and highly concentrated. Within limited timeframes, description becomes the primary means through which the ethnographer compensates for the brevity of immersion. Through detailed and contextually grounded accounts, the researcher reconstructs the field with ‘thick’ description, preserving the immediacy of environments and interactions while giving sustained presence to participants' voices.

Descriptive writing also establishes the foundation for subsequent analysis and interpretation. It ensures that meaning making remains anchored in lived experience rather than drifting into abstracted or decontextualised explanation. In this sense, description performs a dual function within the analytic process: it safeguards the interpretive depth distinctive to ethnography and simultaneously strengthens rigour. By making the empirical world visible, description invites readers into the analytic space, enabling transparency, interpretive accountability and a form of peer engagement with the evidentiary base of the study.

### 3.2 Analysis

When the description phase has been fully developed, the analytic phase formally begins. Wolcott (1994) conceptualises *analysis* as the process of identifying patterns, relationships and categories within data while remaining close to participant lived realities. Analysis, in his view, is about moving beyond mere description to discern structure and meaning, yet without fragmenting the data or stripping it of context. It requires the researcher to search for regularities, consistencies and contrasts that reveal how people organise and make sense of their world. For Wolcott, analysis is not a technical exercise of coding alone but a thoughtful process of pattern recognition that bridges narrative and interpretation.

### 3.3 Interpretation

The process of ethnographic data analysis begins the moment the researcher enters the field, as acts of perception, attention and selection are already interpretive. It unfolds through description, is shaped and refined through writing and is realised in the presentation of findings to readers. According to Wolcott (1994), this analytic movement culminates in *interpretation*. Interpretation is where the researcher moves beyond identifying patterns to explain what those patterns mean within their cultural and theoretical contexts. Interpretation is not simply summarising results but involves constructing meaning and offering insight into the human experience under study. It requires the ethnographer to ‘make sense’ of the data to posit the underlying beliefs, values and organisational principles that shape participants' actions and worldviews. Through interpretation, the researcher conveys to readers not only *what* people do and *how*, but *why* they do so, situating the findings within broader cultural frameworks and scholarly discourse.

### 3.4 Evaluating Wolcott's Framework Through Richardson's Criteria

Richardson's (2000) five evaluative criteria provide a useful lens for assessing the quality of ethnographic analysis. Richardson's (2000) five evaluative criteria are not recent; they remain highly relevant for assessing ethnographic work (Table 1). Revisiting these criteria is particularly timely in contemporary focused ethnography as analytic practice increasingly emphasises structured procedure. General qualitative criteria often prioritise clarity of analytic procedure, thematic saturation and methodological transparency. These dimensions contribute to rigour, yet they do not fully capture the distinctive commitments of ethnography. Richardson's framework therefore provides a lens through which ethnographic analysis can be evaluated in a manner consistent with its methodological nature.

**TABLE 1:** Integrating Richardson's evaluative criteria and Wolcott's analytic framework in ethnographic analysis.

Criterion	Core evaluative focus	Corresponding stage (Wolcott)	Relevance to ethnography
Expression of reality	Does the account convincingly represent lived complexity?	Description	Thick description preserves ambiguity, texture and contextual integrity, ensuring that ethnographic accounts remain grounded in participant lived worlds before abstraction occurs.
Aesthetic merit	Is the work well written, coherent and engaging?	Description	Writing is treated as analytic practice; coherence and structure shape how meaning is conveyed and understood.
Substantive contribution	Does the study deepen understanding of social life?	Analysis; interpretation	Analysis identifies significant patterns and relationships, while interpretation situates these within broader cultural and theoretical contexts, generating explanatory insight rather than thematic listing.
Reflexivity	Does the researcher acknowledge positionality and interpretive role?	Interpretation	Interpretation makes visible the researcher's analytic judgement and acknowledges the situated production of knowledge.
Impact	Does the work move readers intellectually or emotionally?	Description; interpretation	Evocative description moves readers intellectually and emotionally, prompting reflection, reconsideration of assumptions and at times inspiring dialogue or action. Through interpretation, ethnography moves beyond immersion to provocation. By articulating cultural and theoretical meaning, interpretation challenges readers to reconsider taken‐for‐granted assumptions, reframe social realities and engage critically with broader structures.

Richardson (2000) proposes five criteria for evaluating ethnographic work: substantive contribution, aesthetic merit, reflexivity, impact and expression of reality. Substantive contribution concerns whether the study meaningfully advances understanding of social life, offering insights that extend beyond surface description. Aesthetic merit refers to the quality and coherence of the writing, recognising that ethnography is not only analytical but also narrative, and that the clarity and structure of the account shape how meaning is conveyed. Reflexivity requires the researcher to acknowledge their positionality, interpretive role and the conditions under which knowledge is produced. Impact considers whether the work moves readers intellectually or emotionally, prompting reflection, dialogue, or reconsideration of taken‐for‐granted assumptions. Expression of reality asks whether the account convincingly represents the complexity, ambiguity and lived texture of participants' worlds. Together, these criteria foreground the interpretive and representational responsibilities of ethnography, positioning quality as both methodological and humanistic.

When viewed through Richardson's lens, Wolcott's (1994) three‐stage framework reveals a structural alignment between analytic process and evaluative standards. The descriptive stage directly supports the criterion of expression of reality. By requiring the construction of a rich, coherent account before abstraction, description preserves contextual integrity and enables readers to enter the participants' lived world. This stage also contributes to aesthetic merit, as narrative coherence and textured portrayal are integral to effective ethnographic writing. The analytic stage aligns closely with substantive contribution. Rather than merely listing themes, analysis in Wolcott's sense seeks to identify meaningful patterns and relationships that illuminate social organisation. Because analysis remains grounded in the descriptive account, substantive claims emerge from contextual depth rather than detached categorisation. The interpretive stage addresses both impact and reflexivity. Interpretation extends beyond pattern recognition to articulate cultural and theoretical meaning, positioning the researcher's interpretive judgement transparently. In doing so, it enables ethnography to resonate intellectually and emotionally while acknowledging the situated nature of knowledge production.

To clarify how the proposed approach relates to existing methodological guidance, Table 2 positions key analytic frameworks used in focused ethnography and highlights the distinctive contribution of the approach proposed in this paper.

**TABLE 2:** Operationalisation of Wolcott's analytic framework in this study.

Step	Purpose	Data sources	Specific analytic activities
Description	To construct coherent portraits of each participant while remaining close to their lived experiences and contextual background.	Observational fieldnotes; informal conversations recorded during participant observation; in‐depth interview transcripts.	Prepare and compile all relevant data. Immersion into data to develop a holistic understanding of each participant. Create an outline for each vignette using the Sunrise Model (Leininger's Culture Care Theory). Organise descriptive material into sections (e.g., migration history, living context, religion, social networks and health practices). Write narrative vignettes using selected observational and interview data. Revise the narratives to ensure clarity, coherence and fidelity to participants' voices. Conduct peer review of narratives within the research team.
Analysis	To identify patterns across cases in health beliefs, health behaviours and perceived support needs.	Ten narrative portraits constructed from participant observations and interviews.	Review and re‐read the 10 participant narratives to become familiar with patterns emerging across cases. Conduct cross‐case comparison to identify similarities, contrasts and recurring experiences among participants. Perform manual inductive coding following the five‐step process outlined by Creswell and Creswell (2018). Cluster codes into sub‐themes reflecting recurring beliefs, behaviours and perceived support needs. Group sub‐themes into broader themes representing shared experiences across participants.
Interpretation	To explain the cultural meaning underlying the patterns identified in the analysis stage.	Emergent themes; participant narratives; researcher field reflections; relevant theoretical and empirical literature.	Review themes generated during the analysis stage. Re‐examine participant narratives and fieldnotes to ensure interpretations remain grounded in lived experiences. Examine how cultural values, migration histories and social environments shape health beliefs, illness behaviours and perceived support needs. Compare emergent themes with Leininger's Theory of Culture Care and relevant ethnographic and healthcare literature. Develop theoretical explanations on the link between cultures and chronic disease.

## 4 The Application of Wolcott's Framework in a Focused Ethnography

In this section, we explicate how we applied Wolcott's framework for transforming qualitative data in our focused ethnographic study, supported by detailed explanations and examples.

### 4.1 The Focused Ethnographic Study

#### 4.1.1 The Study

This methodological discussion paper draws on data from a doctoral research project approved by the Edith Cowan University Human Research Ethics Committee (Approval No. 21768). The study was designed as a focused ethnography to explore how culture influences health beliefs, health behaviours and perceived support needs among older Vietnam‐born Australians living with chronic disease in Western Australia. This study was conducted in Australia, a highly culturally and linguistically diverse country in the world and hosts a large Vietnamese migrant community (Department of Home Affairs 2018). Evidence indicates that older Vietnam‐born Australians experience a higher burden of chronic disease and poorer quality of life compared to both Australian‐born older adults and other migrant groups (Tran et al. 2013). While existing literature highlights the link between culture and chronic disease management (Payton et al. 2021); there remains limited understanding of how cultural beliefs and practices shape health behaviours, perceptions and support needs in this population.

#### 4.1.2 Research Methodology

The rationale for using focused ethnography in this study was based on the nature of the research question, the theoretical framework, the review of related literature, the researcher's background and the context. Focused ethnography was chosen as the appropriate approach that allowed the researcher to uncover within a social and cultural context through a thick description and close interaction and conversation with members of the specific group of people (Knoblauch 2005). This study followed the six fundamental tenets of focused ethnography including the conceptual orientation of a single research, focus on a definite community, involvement of a small number of participants, problem‐focused and context‐specific and episodic participant observation (Higginbottom et al. 2013). In specific, this study was concerned with a specific cultural group: 12 older Vietnamese people, in which there were two couples, who were born in Vietnam and had migrated to Australia during their adulthood and who were living with chronic disease.

#### 4.1.3 Data Sources

Data sources for this study included fieldnotes and verbatim transcripts collected over approximately 8 months of fieldwork through participant observation and in‐depth interviews. A total of 142 h of participant observation were conducted across multiple settings to document everyday activities through which older Vietnam‐born Australians managed their chronic conditions. The principal researcher participated in daily activities and social events to understand participants' life patterns, cultural practices and health‐related behaviours (Dewalt and Dewalt 2011). Observation followed descriptive, focused and selective phases, allowing the researcher to first gain a holistic understanding of participants' environments and subsequently observe specific practices related to chronic disease self‐management, such as dietary practices, physical activity, religious engagement and social interaction. Informal conversations were held throughout the fieldwork to clarify observations and explore emerging insights (Dewalt and Dewalt 2011). Fieldnotes were recorded in a research diary, capturing both descriptive accounts and reflexive reflections that informed the analytic process.

In addition, in‐depth interviews were conducted to explore participants' beliefs about chronic illness and how these beliefs shaped their self‐management practices and perceived support needs. Interviews also allowed the researcher to revisit and clarify emerging interpretations to ensure that the findings accurately reflected participants' perspectives.

### 4.2 Applying Wolcott's Framework for Data Transformation

It is important to note that analysis began during fieldwork and unfolded as an iterative process that shaped and refined ongoing data collection. The formal application of Wolcott's (1994) analytic stages: description, analysis and interpretation occurred after fieldwork was completed. Guided by Wolcott's framework, the data analysis in this study progressed from raw data to descriptive vignette, pattern recognition and interpretation. The section below outlines how these stages were operationalised during the formal phase of analysis.

#### 4.2.1 Description

The first stage of analysis involved transforming raw ethnographic data into descriptive vignettes. Data used at this stage included observational fieldnotes and verbatim expressions recorded during informal conversations and in‐depth interviews. The purpose of this step was to construct a coherent portrait of each participant while remaining close to their lived experiences. Each vignette situated the individual within their broader context, including living circumstances, family and social relationships, migration history and everyday practices related to managing chronic conditions. This level of description allowed readers to understand participants within their social and cultural contexts before any analytic abstraction was introduced. These materials were synthesised to produce 10 narrative portraits representing 12 older Vietnam‐born Australians living with chronic disease. These portraits later formed the first part of the findings chapter of the doctoral thesis.

A key methodological consideration during this phase was balancing two purposes of description: faithfully representing each participant's lived experience while organising the narratives in ways that would support subsequent cross‐case analysis. To address this, participant stories were structured using the Sunrise Model within Leininger's Theory of Culture Care. This framework helped organise descriptive material without imposing analytical interpretation.

#### 4.2.2 Analysis

Once the descriptive phase was completed, we moved to the second stage of analysis to identify patterns across cases. Specifically, we conducted inductive thematic analysis across 10 case studies following the five‐step outline by Creswell and Creswell (2018) to identify patterns in health beliefs, health behaviours and perceived support needs among older Vietnam‐born Australians living with chronic disease. Manual coding was conducted and progressively clustered into sub‐themes and broader themes relevant to the research questions. Leininger's theory of Culture Care was let out in this step to ensure that emerging patterns were not constrained by prior assumption (Nguyen et al. 2022). The themes generated through this analysis formed the second findings chapter of the doctoral thesis.

#### 4.2.3 Interpretation

Once thematic patterns were identified, we moved to the third stage of analysis: interpretation. At this stage, the aim was to move beyond thematic aggregation to examine what these patterns meant within their broader cultural and social contexts. Interpretation focused on understanding how culture shaped health beliefs, illness behaviours and perceived support needs among older Vietnam‐born Australians living with chronic disease.

Emergent themes were examined in relation to participants' cultural values, migration histories and social environments to explore how these factors influenced their approaches to illness and health care. The themes were then compared and contrasted with Leininger's Theory of Culture Care Diversity and Universality and with the broader ethnographic and health‐care literature. Through this process, Leininger's theory was revisited in the Australian context, where its applicability has often been questioned on theoretical rather than empirical grounds. By grounding interpretation in the lived realities of Vietnamese migrants in Australia, this stage extended the analysis beyond thematic patterns to cultural explanation. Interpretation therefore synthesised participants' lived experiences, the researcher's emic and etic perspectives and theoretical insights to illuminate the interplay between culture, migration and chronic disease management.

Table 3 explicates how Wolcott's (1994) framework was enacted in this study, outlining specific analytic actions undertaken and the resulting outputs in each stage.

**TABLE 3:** Strategies to enhance the quality of this focused ethnography.

Research stage	Strategy
Research design	Comprehensive review of relevant literature to inform the research focus and design.
	2 Application of a theoretical framework to guide the study design and analytical orientation.
	3 Development of an explicit research proposal outlining methodological procedures.
	4 Peer review of the research design by internal and external experts prior to data collection.
Data collection	5 Prolonged engagement in the field (approximately 8 months).
	6 Triangulation of multiple data sources and collection methods, including participant observation, in‐depth interviews and informal conversations.
	7 Regular research team discussions to review field experiences and emerging insights.
	8 Iterative analysis conducted during data collection to refine observations and interview focus.
	9 Member checking: participants were invited to review and verify interim interpretations at the end of the data collection period.
Data analysis	10 Narrative development guided by the study's theoretical framework and research objectives.
	11 Coding and analytic interpretation conducted independently by two researchers.
	12 Use of verbatim participant quotations and observational fieldnotes to support emerging themes and interpretations.
	13 Analytic memo writing throughout the data analysis process to document interpretive decisions.
Interpretation and presentation	14 Provision of rich contextual descriptions of participants and the research setting.
	15 Integration of findings with relevant literature and theoretical perspectives during interpretation.
	16 Thick description of the research context, design, participants and findings to support transferability.
	17 Presentation of findings through both narrative portraits (participant stories) and thematic synthesis to integrate interpretive depth with analytic clarity.

### 4.3 The Quality of This Focused Ethnography

The quality of this study was strengthened through the combined use of Richardson's five criteria (2000) evaluative criteria for ethnography and Lincoln and Guba's (1985) criteria (credibility, dependability, confirmability and transferability) for qualitative research. While Richardson's framework guided consideration of the interpretive and representational quality of the ethnographic account, Lincoln and Guba's criteria informed strategies to enhance the methodological rigour of the research process. Together, these complementary lenses enabled the study to balance systematic rigour with the interpretive commitments of ethnography. The strategies applied to enhance the quality of the study are presented in Table 4.

**TABLE 4:** Positioning the proposed analytic approach in relation to existing methodological guidance in focused ethnography.

Framework author	Primary contribution	Approach to data analysis	Limitations for focused ethnography	Positioning the proposed approach within existing methodological frameworks
Knoblauch (2005)	Conceptualises focused ethnography as a distinct methodological approach characterised by a defined research focus, short‐term fieldwork and intensive data generation	Emphasises data intensity and focused observation but provides limited detail on analytic procedures	Provides limited guidance on how ethnographic data should be analytically transformed beyond general qualitative practices	Clarifies the analytic commitments of focused ethnography and provides a structured pathway for transforming data through description, analysis and interpretation
Higginbottom et al. (2013)	Provides practical guidance for conducting focused ethnography in healthcare contexts	Recommends thematic approaches and general qualitative analytic procedures	Offers limited discussion of how analytic processes preserve ethnography's interpretive and narrative character	Demonstrates how Wolcott's staged analytic framework can preserve contextual richness while supporting systematic analysis
Saldaña (2016)	Provides systematic coding techniques widely used in qualitative analysis	Emphasises coding cycles, categorisation and theme development	Coding‐focused approaches may fragment contextual meaning when applied uncritically to ethnographic data	Repositions coding within a broader ethnographic analytic process that integrates narrative description and cultural interpretation
Wolcott (1994)	Proposes a three‐stage analytic process: description, analysis and interpretation	Emphasises progressive transformation of qualitative data while preserving narrative integrity	Not specifically articulated for focused ethnography or linked to contemporary expectations of methodological rigour	Re‐articulates Wolcott's framework for focused ethnography and demonstrates how it supports both interpretive depth and analytic transparency
Proposed approach (this paper)	Clarifies analytic commitments in focused ethnography	Integrates Wolcott's analytic stages with Richardson's evaluative criteria	Addresses the tension between systematic rigour and interpretive depth in contemporary ethnographic analysis	Provides a coherent analytic pathway that balances narrative storytelling, systematic analysis and interpretive explanation

#### 4.3.1 Researcher Reflexivity

This project forms part of the principal researcher's PhD study. Originally from Vietnam and now living in Australia, the researcher's personal and professional background shaped both the conduct and interpretation of this study. With experience working as a personal caregiver, a nurse and educator in cultural and linguistic contexts, she brought an informed sensitivity to the cultural and social dimensions of health.

In addition, having lived and worked across different cultural contexts, the researcher's understanding of culture was shaped by both insider and outsider perspectives. As a Vietnamese‐born woman, she shared cultural values, language and life experiences with participants, which fostered familiarity, trust and openness during fieldwork. At the time of the study, the researcher was a 31‐year‐old woman while most participants were older adults. Within Vietnamese culture, respect for elders is strongly emphasised, and this age difference required the researcher to remain attentive to culturally appropriate ways of communication and interaction. Her cultural familiarity enabled her to engage participants respectfully and maintain trusting relationships throughout the fieldwork.

The researcher's ability to speak Vietnamese was particularly important, as many participants had limited English proficiency. Communicating in their native language allowed participants to express their experiences more comfortably and enabled richer conversations that may not have been possible in English. This linguistic connection strengthened rapport and facilitated deeper exploration of participants' beliefs, experiences and everyday practices surrounding chronic disease management.

At the same time, the researcher's relocation to Australia, exposure to Western health systems and engagement with multicultural communities positioned her simultaneously as an insider and outsider. This dual positioning enabled her to observe Vietnamese cultural practices with familiarity while also reflecting on them through a comparative lens. Her reflections on gender roles, expectations and food practices across societies also informed her interpretation of participants' experiences. Exposure to different dietary norms and health beliefs helped her recognise both the distinctiveness and universality of cultural practices surrounding chronic disease management.

Throughout the research process, she maintained a reflexive stance, documenting her assumptions, emotions and evolving understanding in fieldnotes and analytic memos. This reflexivity allowed her to remain critically aware of how her positionality shaped data collection, interpretation and representation of participants' voices.

## 5 Practical Considerations

Applying Wolcott's framework in contemporary ethnographic research presents both opportunities and challenges. While the framework offers a means to balance storytelling with scientific rigour, researchers must navigate several practical and conceptual tensions. The descriptive phase, though essential, can easily become overwhelming. Lengthy narratives may obscure analytic clarity, and novice researchers often struggle to determine how much detail to include. Using theoretical concepts and research questions as guides can help maintain focus, ensuring that stories remain rich yet purposeful. Peer review of descriptive drafts can further assist in balancing vividness with analytical coherence. Time constraints in focused ethnography also present challenges. Conducted within short, intensive fieldwork periods, this form of ethnography demands prioritising depth over breadth. Developing detailed portraits of a smaller number of participants, supported by triangulation of data sources such as observations, interviews and documents, can help preserve analytic depth despite limited time. Ethnographers must also balance the dual demands of *science and art*. They are expected to produce work that satisfies contemporary standards of transparency and rigour while preserving the narrative and emotional power that make ethnography distinctive. This requires what Wolcott and Richardson might call ‘dual literacy’ (Richardson 2000; Wolcott 1994). It is the ability to move fluently between systematic coding and rhetorical storytelling. Presenting findings through both thematic synthesis and participant stories allows ethnographers to meet academic expectations while maintaining authenticity. Applying Wolcott's framework also demands a wide skill set, from evocative writing to systematic coding and theoretical interpretation. For emerging researchers, this can be daunting. Developing these literacies incrementally beginning with descriptive writing, progressing to cross‐case analysis and culminating in interpretive synthesis provides a scaffolded pathway. Collaborative research teams can also distribute these competencies across members, enriching both process and outcome. A further tension arises from contemporary notions of qualitative rigour, particularly in applied health research. Ethnographers may encounter scepticism if their findings appear too ‘literary’ or insufficiently systematic, while over‐reliance on coding frameworks risks eroding ethnography's distinctive identity. Transparency offers a solution: clearly articulating how description, analysis and interpretation were integrated, and how narrative and coding were used in tandem.

On a personal note, engaging with Wolcott's framework demanded perseverance, reflexivity and intellectual maturity. The process was often challenging, requiring the courage to balance creativity with the contemporary expectations for analytical rigour and productivity. Yet it was within this tension that the most profound learning occurred. Through grappling with the interconnections among description, analysis and interpretation, I learned not only how to describe and explain culture, but also how to think more reflexively and critically about the process of ethnographic inquiry. This journey reinforced that conducting ethnography is not merely the application of analytic procedures, but the development of intellectual and interpretive capacities. Ultimately, the value of engaging deeply with this process lies in the growth it fosters in researchers and in the richer, more compassionate understanding of human experience that ethnography makes possible.

From these reflections, several practical lessons emerge for researchers applying Wolcott's framework in focused ethnography:

1. Description must remain purposeful: While storytelling remains central to ethnography, descriptive narratives need to be guided by the research questions and theoretical or conceptual frameworks to ensure that accounts remain analytically meaningful rather than purely descriptive
2. Depth can be achieved within focused fieldwork: Even in limited field engagement, analytic depth can be preserved by developing detailed participant portraits and triangulating multiple sources of data.
3. Ethnographers require dual analytic literacy: High‐quality ethnographic analysis requires the ability to move between systematic analytic procedures and narrative storytelling to balance rigour with interpretive depth.
4. Transparency strengthens ethnographic rigour: Clearly articulating how description, analysis and interpretation are integrated helps demonstrate methodological rigour while preserving the distinctive interpretive character of ethnography.

## 6 Conclusion

Our article offers an approach to analysing ethnographic data that preserves the methodology's distinctiveness while meeting contemporary scientific standards of quality. Drawing on Wolcott's three‐stage framework for transforming qualitative data: description, analysis and interpretation, we demonstrated how focused ethnographic analysis can integrate narrative storytelling with systematic analytic procedures. By explicitly articulating how these stages interact, the paper provides a practical pathway for researchers to conduct ethnographic analysis that maintains interpretive depth while meeting expectations for methodological transparency and rigour. We argue that the raw, organic story of each individual, when written purposefully and theoretically, enhances both the quality and credibility of ethnographic work. Ethnography, at its heart, is both art and science: a way of seeing, feeling and understanding human experience in its living context. In today's research environment, where efficiency and productivity are often prioritised, the depth and distinctiveness that define ethnography are at risk of being lost. To remain meaningful and credible, ethnography must be held to what Laurel Richardson calls ‘high and difficult standards’ that value reflexivity, creativity and authenticity as much as precision and coherence. Upholding these standards is demanding, but it is through this challenge that ethnography retains its integrity and continues to offer what no other method can: a way of knowing the world that is both scientific and humanistic.

## Author Contributions

**Thi Ngoc Minh Nguyen:** conceptualisation, design, manuscript development and revision. **Gordana Dermody:** critical review of the manuscript. **Rosemary Saunders:** critical review of the manuscript. **Lisa Whitehead:** overall supervision and critical review of the manuscript.

## Funding

The authors have nothing to report.

## Ethics Statement

The authors have nothing to report.

## Conflicts of Interest

The authors declare no conflicts of interest.

## Data Availability

Data sharing not applicable to this article as no datasets were generated or analysed during the current study.
